# Impacts of the North American signal crayfish (*Pacifastacus leniusculus*) on European ecosystems

**DOI:** 10.1186/s12302-015-0065-2

**Published:** 2015-12-09

**Authors:** Susanne Vaeßen, Henner Hollert

**Affiliations:** Department of Ecosystem Analysis, Institute for Environmental Research, ABBt – Aachen Biology and Biotechnology, RWTH Aachen University, Worringerweg 1, 52074 Aachen, Germany

**Keywords:** Signal crayfish, Benthos, Fish species, Biological invasion

## Abstract

As a vector of the crayfish plague (*Aphanomyces astaci*), invasive crayfish pose a major threat to endemic crayfish species in Europe. But do they affect whole ecosystems and fish species as well? A comprehensive review was done using online search engines on current literature to elucidate possible crayfish effects. It showed that they have the potential to decimate benthic invertebrate populations as well as submerged plants—the first a necessary food source, the second an important part of the habitat of fish, functioning as hiding space for their fry as well as their prey. Crayfish are suspected to act as bioturbators as well, by influencing preconditions to certain algae and animals while sorting through the substrate of a river. Their long-term effects on fish so far are inconclusive. Studies on this matter showed no effect, selective impact on fish that share prey with the crayfish, as well as significantly negative effects on fish in general. In shorter examinations, invasive crayfish have proven to displace fish from shelters, putting them at a higher risk for predation. Moreover, comparisons to native crayfish species showed that these had less negative effects on fish—due to lower consumption and reproduction rates and population densities.

## Background

In many countries of Europe, the invasive signal crayfish (*Pacifastacus leniusculus*) has become a huge problem for endemic crayfish species [1–4] transmitting the crayfish plague (*Aphanomyces astaci*), a water mold that eradicates whole populations of European species when introduced into a water body—with no effect on the invaders [5, 6].

Since signal crayfish reach impressive densities in short time, the question arises whether they affect not only endemic crayfish but the ecosystem as a whole. Preliminary to a study focusing on that question, we did a literature review on the topic to find out which effects of the crayfish have already been discovered. We were using the keywords “signal crayfish” and “*Pacifastacus leniusculus*” in combination with “effects”, adding “fish” or “ecosystem”, respectively, in a second and third search run in Google Scholar. The results are presented in this paper.

## General crayfish feeding behavior

Crayfish are generally omnivorous but preferentially predatory animals. If macrobenthos is not available in sufficient quantities, they consume huge amounts of less attractive food like macrophytes. These are an important piece of littoral habitats, functioning as spawning grounds and hiding places for juvenile fish and their prey [7].

Examinations of signal crayfish’s gut contents showed vascular detritus, filamentous green algae, crayfish fragments and larvae of Chironomidae and Ephemeroptera to be the main parts of its diet throughout the year. Direct predation of fish as well as cannibalism occurred. *Pacifastacus leniusculus* mainly feeds at night. The estimated ration of a day ranged from 0.22 to 6.02 % of wet body weight with bigger rations in adult animals and during summer and autumn [8].

Two years earlier, a study on the same signal crayfish population gave an idea of the biomass consumed by them. In the examined river section, densities of 10–15 animals per m^2^ were found with a biomass of 53–61 g/m^2^ over 1 year, while density in optimal areas of the habitat could reach considerably higher numbers [9].

Taking these studies as a basis, one can conclude that a signal crayfish will consume an average 3.12 % of its body weight daily. In a river section of 100 m, a width of 4 m and an average crayfish mass of 57 g/m^2^, this would mean a consumption of 260 kg of macrobenthos and macrophytes per year or even considerably more depending on conditions. This magnitude of influence should become apparent in the general ecology of a water body.

## Crayfish effects on ecosystems

So how does biocenosis react to the presence of those preferably predatory omnivores?

Dorn and Wojdak [10] found decreasing numbers in young-of-the-year residential fish after crayfish had been introduced to a pond in which they fed extensively on fish spawn. As a result, zoo plankton biomass increased while O_2_ concentrations decreased, apparently mediated by unfavorable ratios of respiration and primary production. Filamentous green algae disappeared quickly while blue algae of the genus *Gleotrichia* (a less coveted food source) finally dominated the community. *Chara vulgaris* and vascular macrophytes, which covered 34 % of the area in control ponds, could not develop. Periphyton-consuming polliwogs and gastropods were significantly reduced or could not be found at all [10].

A decrease of 70 % of benthic invertebrates and 90 % of periphyton biomass could already be observed at a crayfish density of only 1.8 per m^2^ [11].

Crayfish also might have other than just trophic effects on ecosystems. Dorn and Wojdak [10] for example, suspected effects on phytoplankton due to bioturbation.

Foreign crayfish species introduced to ecosystems that are not laid out for their presence can become invasive. They show different reproduction rates, behavior and feeding habits than residential species and might have a considerably stronger impact on the system [12–16].

A study conducted in Swedish ponds revealed a decrease of biomass, vegetated ground area and diversity of macrophytes with increasing density of invasive signal crayfish. The composition of plant species was influenced as well. Apart from that, decreasing abundance in herbi- and detritivorous invertebrates could be examined, while predatory invertebrates only decreased in ponds with low pH. The invertebrate community was increasingly dominated by sediment-dwelling species. In addition to that, the organic portion of the sediment decreased [17].

Similar results were obtained using in situ cages in a pond populated by signal crayfish. Natural signal crayfish densities had a significantly negative impact on predatory invertebrates and a very strong one on aquatic snails. The snails’ decrease led to an increase of periphyton biomass due to reduced grazing. Herbivorous tadpoles slightly increased, but the percentage of surviving frogs was smaller in crayfish cages than in controls—probably due to predation of injured tadpoles, which often suffered tail injuries in crayfish cages. Macrophyte cover decreased by consumption as well as mere dissection [18].

In Swedish experiments over a period of 1 month, gut contents of surviving crayfish were examined after the time of exposure. Two different crayfish densities were kept in cages with twenty young trouts (*Salmo trutta*). Detritus and animal constituents proved to be the main food sources of crayfish in these experiments. Algae and macrophytes only played a subordinate role. Crayfish did not have any influence on the survival rate of trout, which was positively related to streaming velocity instead. However, negative effects on biomass and diversity of invertebrates (especially predatory species) were found again. Epilithic algae increased with crayfish density—probably due to improved conditions of lighting and nutrition since active crayfish resuspend and/or remove detritus and aging algae cells during periods of low flow velocities. The researchers predict a decrease of macroinvertebrate diversity in invaded communities as well as elimination of susceptible predatory invertebrates. In streams that carry huge amounts of sediment or organic material, high crayfish densities will increase benthic algae production by bioturbation [19].

Crawford et al. [20] examined the effect of a newly introduced signal crayfish population on the invertebrate community in the River Clyde (Scotland). River sections populated by crayfish were compared to similar sections without colonization. Reduction of invertebrates could be verified in this study. Their population density in crayfish sections proved to be only 60 % of the density in non-populated sections. Biodiversity also decreased in areas with crayfish population [20].

The littoral food web of a marsh was taken into focus in a cage experiment in Japan. In addition, effects of differently sized crayfish were examined. Big crayfish (>30 mm carapax length) quickly eliminated aquatic macrophytes by mechanic destruction while similar effects of smaller crayfish were only noticeable after a longer period of time. Biomass of benthic algae was reduced in the presence of big crayfish but only marginally influenced by small ones which leads to the conclusion that big crayfish act as bioturbators. In this study, diversity of invertebrates was almost halved in the presence of big crayfish, which is probably due to the reduction of rare taxa. According to the Japanese study, possible influences of crayfish on invertebrates are: the predation of big susceptible taxa like caddisflies and predatory invertebrates, the mechanic destruction of macrophytes and associated reduction of invertebrate’s microhabitats, as well as increased emigration of invertebrates due to bioturbation and/or relief in predation and competition for small invertebrates as a result of the reduction of their enemies/competitors.

The functional roles of signal crayfish in an ecosystem stayed the same during their ontogenetic development but the magnitude and rate of their influences intensified with growing size [21].

## Differences between effects of native and invasive crayfish

Whether invasive crayfish have more negative influences than native ones has been investigated specifically in two studies.

In Sweden, Nyström and Strand [22] compared grazing behavior of native noble crayfish and invasive signal crayfish on seedlings and adult macrophytes. Seedlings and adult plants of tule (*Scirpus lacustris*) and broad-leaved pondweed (*Potamogeton natans*) as well as *Chara vulgaris* were offered to both species. *Chara vulgaris* was preferred over other plant species. Signal crayfish consumed significantly more *Chara* than noble crayfish—especially at higher temperatures. Results indicate that signal crayfish are the more voracious grazers with a larger negative impact. *Chara* seems to be particularly vulnerable since it is preferred by crayfish and the genus contains a large number of rare species. The occurrence of signal crayfish thus harbors a higher risk of reduction or even extinction of submersed plants than that of the native and less voracious noble crayfish [22].

Three years later Nyström et al. compared effects of both crayfish species on a complete benthic food web. They imitated a pond shore habitat in large plastic basins filled with natural densities of macrophytes, invertebrates and either signal or noble crayfish or as crayfish-free controls. Results were evaluated after two summer months. With regard to the overall impact on the ecosystem, similar findings as in previous research could be found. Crayfish took in most of their carbon from invertebrates and less from primary producers and had no effect on biomass of predatory invertebrates which mainly consisted of active swimmers. They had a strong impact on grazers and an indirect positive effect on periphyton on the substrate, probably due to the reduction of grazing snails. Crayfish grazed selectively on macrophytes and reduced the biomass of *Chara* while *Elodea* was less affected.

Again, the overall impact of the exotic signal crayfish proved to be greater than that of the native noble crayfish [23]. Since consumption rates are higher in signal crayfish, it is to be expected that this species will have a stronger impact on an ecosystem.

## Effects on fish

### Long-term field studies

So far, three studies dealt with the long-term impacts of invasive crayfish, only two of which with the main focus on fish—and they all came to different results.

The first long-term field study (4 years), which exclusively focused on the reactions of fish stock on signal crayfish, was conducted in Sweden by Degerman et al. [24]. Examination of streams showed no negative effects of signal crayfish on fish. Comparisons of fish densities within stream sites in years with and without crayfish presence revealed no significant impact. Population density of crayfish had no effect either [24].

Wilson et al. [25] examined effects of a rusty crayfish (*Orconectes rusticus*) invasion on an entire ecosystem in a lake in the USA, lasting 19 years at the time of examination, during which the crayfish had spread along the entire littoral zone. In contrast to Degerman et al., they found a decrease in fish species that shared prey with the crayfish, while piscivorous fish showed no such reactions. Those selective effects can be easily explained by changes in the ecosystem. Snails were partially reduced from >1000 to only <5 animals per square meter. Average numbers of Odonata, Trichoptera and Amphipoda decreased significantly. Native crayfish disappeared almost completely, although overall crayfish occurrence increased due to the high density of rusty crayfish. Diversity of submerged macrophytes decreased up to 80 % in some places. This long-term study showed a different result using the same approach at least with regard to fish species in direct food competition with the crayfish [25].

The third long-term study addressed the impact of signal crayfish on fish of the water column. Since the increasing spread of the signal crayfish in England causes concern for the native trout (*Salmo trutta*) and salmon (*Salmo salar*), Peay et al. examined the head water of a Yorkshire stream in which native white-clawed crayfish (*Austropotamobius pallipes*) were gradually displaced by *P. leniusculus*. Densities of fish and both crayfish species were compared over a period of 2 years. The study revealed a significantly negative correlation between fish and signal crayfish densities. Sample areas with white-clawed crayfish (1–2 crayfish caught per night) had numerous young trout (>47/100 m^2^). Signal crayfish in contrast, did not only reach higher densities (4–8 crayfish caught per night), the populated areas also had less fish (0–18.8/100 m^2^) [26].

### Shorter studies regarding direct competitive behavior

Results of shorter studies draw a clear picture of negative invasive crayfish effects on fish. Competition for shelter and food can be identified as the main reasons for fish decline.

#### Changes in behavior/competition for shelter

Guan and Wiles [27] investigated competition for shelter and predation by crayfish. They focused on interactions between signal crayfish and benthic fish in a British river and discovered a negative correlation between crayfish density and densities of the two most abundant fish species—bullhead (*Cottus gobio*) and stone loach (*Noemacheilus barbatulus*). Population density of benthic fish was lowest in the riffle closest to the original crayfish stocking site and gradually increased up- and downstream with decreasing crayfish density. The hypothesis that crayfish and benthic fish compete for shelter and the fish are predated by crayfish was tested in a flume containing artificial shelters. Fish of either one of the species were kept alone or with crayfish in 3-day cycles for a total of 12 days. Results showed crayfish to be superior to both fish species in shelter occupation. Direct predation was examined by keeping 24 fish of each species either alone or with 36 crayfish in the flume for 10 days at a time. Mortality rates of both fish species were significantly higher when crayfish were present. Crayfish guts contained remains of some lost fish and they were observed to catch fish of both species. In the river, crayfish reached high densities (more than 20 individuals/m^2^ in riffles) and the population was still continuing to spread. A strong reduction, and even local extinctions, of benthic fish might be the outcome [27].

In California, signal crayfish have been associated with reduced growth rates and gut content of Paiute sculpin (*Cottus beldingi*). Light [28] tried to determine their effects on behavior and habitat use of the sculpin. These reduced their use of shelters and pools, switched to microhabitats with higher flow velocities and spent more time with flight behavior if crayfish were present. Crayfish on the other hand, used shelters, pools and low flow velocity habitats more often than sculpin. Both species were mainly active at night. Detailed field studies in the lower reaches of the creek revealed that potential shelters (single exposed rocks) were closely related to total numbers of sculpin and crayfish which leads to the suspicion that the abundance of shelters can have a limiting effect under natural conditions. Therefore, crayfish might increase the predation risk on sculpin by displacing them from shelters and pools and increasing their activity rate. Behavioral changes of sculpins seemed to be at least partially responsible for their reduced growth rate in the presence of crayfish [28].

Bubb et al. [29] were able to demonstrate that competition for shelter occurs more strongly with signal crayfish than with native crayfish. They examined behavioral interactions and competition for shelter between native sculpins and white-clawed crayfish as well as invasive signal crayfish. Although both crayfish species proved dominant to sculpin (sculpin evaded approaching crayfish, left shelters if they were entered by them and rarely swam into shelters occupied by them), signal crayfish were significantly more aggressive than white-clawed crayfish. If sculpins were kept alone, they spent most of the day in shelters (averagely 96 %) which slightly relaxed at night (averagely 60 %). While both species of crayfish reduced the shelter use of sculpins, the fish would share shelters with white-clawed crayfish more often than with signal crayfish. But higher fertility and population densities of the species in comparison to native crayfish might ultimately be even more important than behavioral differences [29].

However, competition for shelter with signal crayfish not only has negative effects on benthic fish but also on fish of the water column. Griffiths et al. [30] showed that signal crayfish displaced juvenile salmon from shelters. The experiments were conducted in winter since salmon become nocturnal if water temperatures drop below 10 °C, increasing competition with the generally nocturnal crayfish. The percentage of sheltering Atlantic salmon was significantly lower if crayfish were present. The percentage of sheltering signal crayfish on the other hand was not influenced by the presence of salmon. If salmon instead of crayfish density was increased, the percentage of sheltering salmon was significantly higher in intraspecific trials than in interspecific ones. Apparently, fish were able to compromise with their own better than with crayfish. It is to be expected that salmon which do not shelter during winter days are highly vulnerable to predation. Therefore, competition for shelter with crayfish could lead to negative effects on the salmon population [30].

#### Competition for food

Effects of food competition between fish and crayfish were studied in the laboratory by Carpenter [31]. Experiments were conducted with the aggressive omnivorous crayfish *Orconectes virilis*, which had invaded the previously crayfish-free Colorado River Basin, and two native fish species—the Gila chub (*Gila intermedia*) and the Flannelmouth Sucker (*Catostomus latipinnis*). Population density of the species was varied in the experiments. Each fish species was tested in separate trials. While growth rates of the Gila chub were mostly affected by intraspecific competition, growth of the Flannelmouth Sucker was more strongly affected by crayfish presence. In contrast, growth rates of crayfish were not significantly influenced by the presence of either one of the two fish species. Carpenter thus found a species-specific influence of food competition [31].

Competition for shelter seems to play a particularly important role between fish and crayfish, apart from food competition and direct predation by crayfish.

## Conclusions

Crayfish definitely have the potential to influence ecosystems—mainly by consumption of macroinvertebrates and plants. Invasive species like the signal crayfish reach higher population densities, have higher consumption rates and spread at a higher pace, which is the main reason for their even stronger negative effects. Even at low population densities, they can lead to decreasing numbers and diversity in benthic invertebrates and macrophytes as well as a shift in species composition. Results on their impact on natural fish populations were inconclusive in long-term studies, showing negative, selectively negative or no impact on fish. Short-term studies, however, were consistently proving a negative effect on fish by competition for shelter and food. The magnitude of their impacts on ecosystems may differ depending on the waterbody type, since streaming water and standing water house different species and communities. Their negative effects on fish in ponds or lakes did not show as strongly in streaming water. Also, the reduction of macrophytes will not show as strongly in streaming water as in ponds, since especially in the higher regions, macrophytes play a subordinate role in the ecosystem of a stream.

To shed more light on the influences of signal crayfish on ecosystems, a study is currently conducted in which the spread of two signal crayfish populations and their impact on fish and macroinvertebrate populations is followed over the course of 3 years. By closely watching certain areas of a stream while they are being populated, the authors hope to find out more about the effects of the invaders.
